# Clinical considerations for the design of PROTACs in cancer

**DOI:** 10.1186/s12943-022-01535-7

**Published:** 2022-03-07

**Authors:** Cristina Nieto-Jiménez, Esther Cabañas Morafraile, Carlos Alonso-Moreno, Alberto Ocaña

**Affiliations:** 1 Experimental Therapeutics Unit, Medical Oncology Department, Hospital Clínico San Carlos (HCSC), Instituto de Investigación Sanitaria (IdISSC) and CIBERONC, 28040 Madrid, Spain; 2 Center for Biological Research, Margarita Salas (CIB-CSIC), Spanish National Research Council, Madrid, Spain; 3 Centro Regional de Investigaciones Biomédicas (CRIB), Universidad de Castilla-La Mancha, 02008 Albacete, Spain; 4 Facultad de Farmacia de Albacete, Universidad de Castilla-La Mancha, 02008 Albacete, Spain; 5 Translational Research Unit, Albacete University Hospital, 02008 Albacete, Spain

**Keywords:** PROTAC, New therapies, Clinical approach, Protein of interest

## Abstract

Degradation of targeted proteins using proteolysis targeting chimeras (PROTACs) has gained momentum. A PROTAC is a bifunctional molecule that consists of three parts: a ligand that interacts with the protein to be degraded, another ligand that binds to an E3 ubiquitin ligase and a linker that connects both. Identification of the right proteins as targets to be degraded and a ligase that is highly expressed in tumors compare with normal tissue is mandatory, as can augment efficacy reducing toxicity. In this article we review the current development stage of PROTACs in cancer to categorize the best PROTAC construction. Targets including BCL2, CDK4 and MCL1 were highly expressed in all tumors; MCL1 was significantly increased in breast cancer and lung adenocarcinoma and CDK4 in colon adenocarcinoma. Degradation of CDK9, AURKA or PLK1, followed by BCL2, MCL1, PTPN11, BRD4, PTK2, showed a high dependency. Most ligases evaluated were not highly present in tumors except for MDM2 in breast, lung, prostate and gastric cancer. In non-transformed tissue MDM2 was the most abundant ligase, followed by cIAP and CRBN, and those with low expression included XIAP and VHL. MDM2 ligase coupled with inhibitors of the targets BCL2, BRD4, CDK9, PLK1 and MCL1 in stomach tumor, and MDM2 with PIK3C3 inhibitors in breast cancer, seems to be the best therapeutic strategy. Our results suggest potential options for the design of PROTACS in specific medical indications.

**Supplementary Information:**

The online version contains supplementary material available at 10.1186/s12943-022-01535-7.

## Introduction

Identification of oncogenic or non-oncogenic druggable vulnerabilities is a main aim in cancer research [1, 2]. Oncogenic processes are mainly produced by structural genomic alterations including mutations, copy number variation or genomic rearrangements [3]. Some of these modifications translate to modified proteins with a gain or loss of function that favors survival or proliferation, among others biological roles [3]. Agents targeting proteins with enzymatic activity have shown clinical benefit like those acting against kinases, including small tyrosine kinase inhibitors or antibodies against membrane receptors. Examples comprise trastuzumab or cetuximab against HER2 or EGFR, or vemurafenib against the BRAF V600 mutated protein [4, 5]. Targeting non oncogenic druggable vulnerabilities have also shown clinical efficacy, for example using drugs acting against epigenetic components, or the tumor microenvironment, or when optimizing synthetic lethality interactions, where a classic example is the use of PARP inhibitors in tumors harboring BRCA1/2 mutations [6–8]. Beyond the druggability properties, inhibition of pan essential genes in tumor cells has shown preclinical and clinical activity, since those genes are involved in relevant biological functions, including cell cycle proliferation or transcription, among many other functions.

One of the main limitations in drug development is the fact that most chemical agents act against the enzymatic activity of proteins, with very limited options for targeting oncoproteins without this activity [8, 9]. Recently, degradation of targeted proteins using proteolysis targeting chimeras (PROTACs), has gain momentum [10, 11]. This family of agents mediate the degradation of a target proteins through its ubiquitination, and subsequent degradation mediated by the proteasome [10, 11]. A PROTAC is a bifunctional molecule that consists of three parts: a ligand that interacts with the protein to be degraded (warhead ligand), a different ligand that binds to an E3 ubiquitin ligase and a linker that connects both ligands [10]. These agents have shown strong potency compared with the counterpart chemical inhibitor and, some of them, have reached the clinical setting, like those developed against the estrogen and androgen receptors, in breast and prostate cancer, respectively [12].

Selection of the right proteins as targets to be degraded is key since the complete depletion of some genes may affect cell viability in oncogenic and non-transformed tissues [10]. This could happen when acting on proteins that are coded by pan-essential genes and their role in normal cells is relevant. In this context, a narrow therapeutic index could limit the development of these agents [9]. Among the different strategies to avoid toxicity in non-oncogenic tissue is the selection of a ligase not expressed in the non-transformed tissue, so no off-tumor effects will be observed. Similarly, the selection of one ligase highly expressed in the tumoral cell type could augment the therapeutic effect.

In this article we review the current development stage of PROTACs in cancer, focusing on the target selected, and the potential effect on cell viability that would have their degradation. Similarly, we will review if the selection of the ligase could reduce the toxicity by no acting on non-tumoral tissue. Our results show that PROTACs against pan-essential genes could have a narrow therapeutic index that would limit their clinical development. On the other hand, the identification and use of ligases for specific tumor tissues become a promising alternative to reduce toxicity and augment efficacy.

## Material and methods

### Search and classification of all designed PROTACs

We performed a search for scientific articles in the PubMed digital repository, specifying "DESIGN PROTAC", in order to identify all PROTACs targets.

We found 156 scientific articles with this search, 92 of those were not reviews and whose described compounds were detailed described. Then, all these compounds were classified according to the protein of interest, the biological function of the target, and the ligase used in the PROTAC.

### Identification of targets and ligases with the greatest potential for the design of new PROTACs

The effect of inhibiting or suppressing these targets in different cell lines was studied using the DepMap portal software tool (https://depmap.org/portal/) (last accessed in July 2021). This tool is used to analyze whether a target is essential for different cell types (common essential) or for a specific group (strongly selective). From the score provided by DepMap (CERES, for CRISPR and DEMETER2, for siRNA), we selected a mean -0.7 score as an arbitrary threshold to choose those genes whose inhibition is closer to being essential. We focused only on those tumors with the highest incidence including breast, colon, lung, gastric and prostate cancer.

Afterwards, with the web tool GEPIA2 (Gene Expression Profiling Interactive Analysis; http://gepia2.cancer-pku.cn/) (last accessed in August 2021) [13], we analyzed the expression (in transcripts per million, TPM) of these targets and ligases in the tumoral tissue of the mentioned five cancer types. We selected those combinations whose TPM was equal to or greater than 32, a value considered medium/high in terms of gene expression [14].

### Identification of possible toxic effects with the use of certain PROTACs

We again used GEPIA2 [13] to study the expression of ligases and targets in different normal tissues. We choose those that exceed the expression of 32 TPM [14].

## Results

### Evaluation of targets used as warhead ligands for the development of PROTACs

**Fig. 1:**
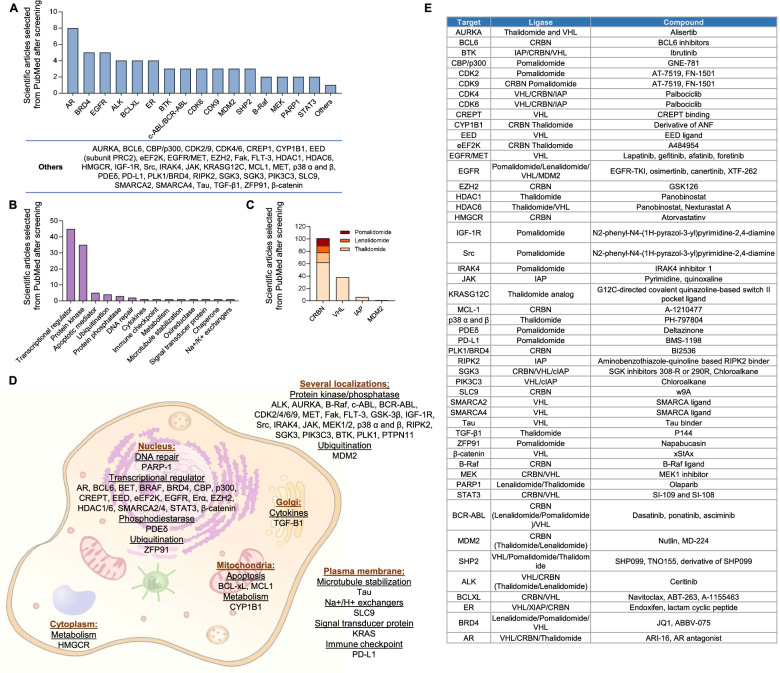
Classification of the number of scientific articles per target (**A**), function of the target (**B**) and the type of ligase ligand (**C**). **D** Representation of the targets and the function in which they are involved. **E** Compounds and ligases linked to each target

We first explored the range of different targets against PROTACs that are currently in preclinical development. Based on a literature searched (Suppl Table 1), as described in material and methods, the most frequent targets for which ligand warheads are under development, included the androgen receptor (AR), followed by BRD4, EGFR and ALK, among others (Fig. 1A). When exploring the type of target for which these drugs were developed, we observed that most of the PROTACs were designed against transcriptional regulators or against proteins with kinase activity (Fig. 1B), followed by a range of targets with different biological roles.

We next evaluated the different ligase ligands used for these agents, being the most frequently used cereblon (CRBN), followed by von Hippel-Lindau tumor suppressor (VHL), and islet amyloid polypeptide (IAP), as shown in Fig. 1C. The biological functions of the full list of targets, included apoptosis (MCL 1 and BCL 2), cell cycle regulation or division (CDK2 and CDK 4; AURKA, PLK1), transcription (CDK9 and BRD4), cell signaling (PI3KC3, PTPN11) or metabolism (HMGCR), displayed in Fig. 1D. Moreover, the name of the specific compound and the type of ligase ligands are shown in Fig. 1E.

### Effect of target/gene knock out of cell survival

**Fig. 2:**
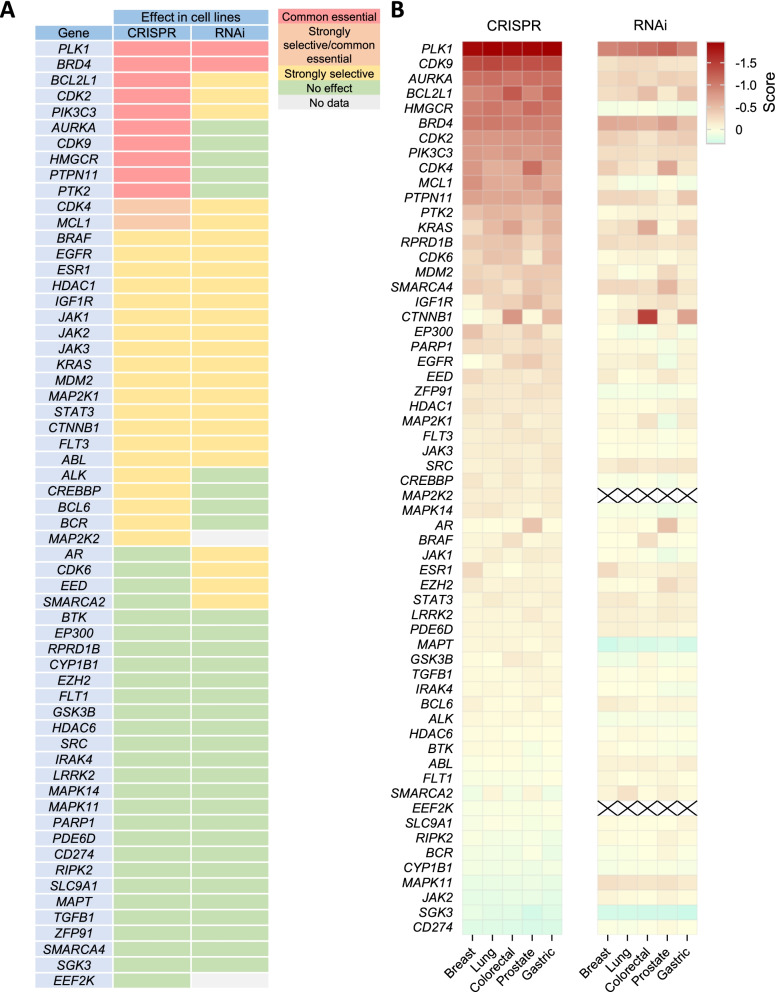
Effect of target silencing on different tumor types. **A** Effect of silencing by both CRISPR and RNAi on different cell lines from several tumor types. Data from DepMap portal. Common essential: A gene which, in a large, pan-cancer screen, ranks in the top X most depleting genes in at least 90% of cell lines. X is chosen empirically using the minimum of the distribution of gene ranks in their 90th percentile least depleting lines. Strongly selective: A gene whose dependency is at least 100 times more likely to have been sampled from a skewed distribution than a normal distribution. **B** CERES and DEMETER2 dependency score of each gene in the five tumors with the highest incidence including breast, lung, colorectal, prostate and gastric cancer. A score of 0 indicates that a gene is not essential, and a score of -1 is comparable to the median of all pan-essential genes

To evaluate if any of the drugs under development use warhead ligands targeting pan-essential genes, we analyzed the effect on cell survival of the downregulation of these genes by siRNA and CRISPR. For this purpose, we used a bioinformatic approach as described in the material and methods section. Figure 2A shows the lists of targets classified as common essential or strongly selective. In order to detect pan-essential genes, we used the CERES and DEMETER2 dependency score of each gene in the five most prevalent tumors: breast, lung, colorectal, prostate and gastric cancer. As shown in Fig. 2B, some genes, such as *CDK9, AURKA* or *PLK1*, followed by *BCL2, MCL1, PTPN11, BRD4, PTK2*, and *MHGCR* or *PI3KC3*, showed dependency. Others included *CDK4* and *CDK2*. Differences were observed between siRNA or CRISPR demonstrating that the complete deletion of the gene induced a more profound effect on cell survival.

### Expression of relevant targets and ligases in selected human tumors

**Fig. 3:**
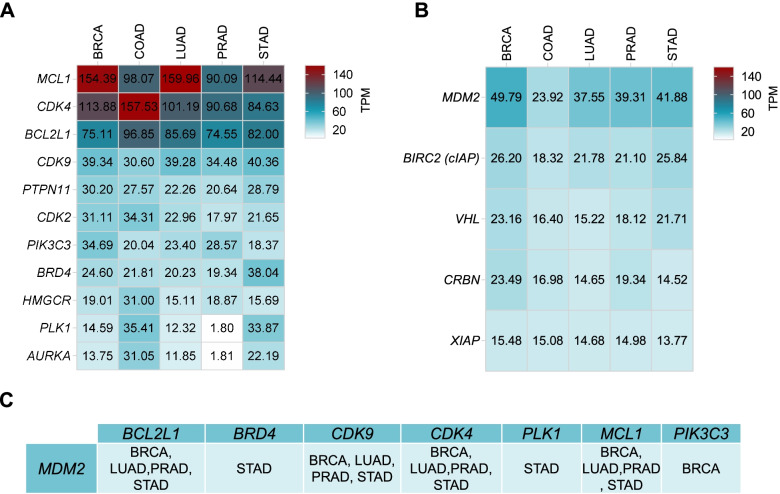
**A** RNA expression in TPM of targets in tumoral tissues with the highest incidence according to GEPIA2. **B** RNA expression in TPM of ligands in tumoral tissues with the highest incidence according to GEPIA2. **C** Targets and ligases with TPM > 32. BRCA: Breast Invasive Carcinoma, COAD: Colon adenocarcinoma, LUAD: Lung adenocarcinoma, PRAD: Prostate adenocarcinoma, STAD: Stomach adenocarcinoma

To get insights into the specific tumors where these targets would be highly expressed, we interrogated TCGA/GTEx in GEPIA2 to evaluate their expression in the previous described tumors. *BCL2, CDK4* and *MCL1* were highly expressed in all tumours studied (Fig. 3A). *MCL1* was significantly increased in breast cancer and lung adenocarcinoma, and *CDK4* in colon adenocarcinoma. *CDK9* was present, to a smaller extent, in breast, lung, prostate and gastric cancer (Fig. 3A). Following this, we analyzed the expression of the ligases observing that most of them were not highly present in these tumors with the exception of *MDM2* in breast cancer, lung, prostate and gastric cancer (Fig. 3B).

Finally, we studied the association between the presence of the target and the ligase with the objective to identify novel indications where PROTACs could be developed. In this context, we identified tumors for which both targets and ligases have TPM expression greater than 32, using this cut off as described in material and methods. We observed that the MDM2 ligase could be used to create warhead ligands against BCL2, BRD4, CDK9, CDK4, PLK1, MCL1 and PIK3C3. Figure 3C shows in which tumor indication MDM2 could be developed for a specific warhead ligand. Of note, stomach adenocarcinoma followed by breast cancer were the tumor types with more upregulation of the targets. In summary, this data describe tumor types were specific PROTACs could be developed, including the warhead ligand and the specific ligase.

### Expression of ligases in non-transformed human tissue

Since the proteins of interests (warhead ligands) and ligases are also present in non-transformed tissues, we decided to study their expression, to identify those less frequently present.

**Fig. 4:**
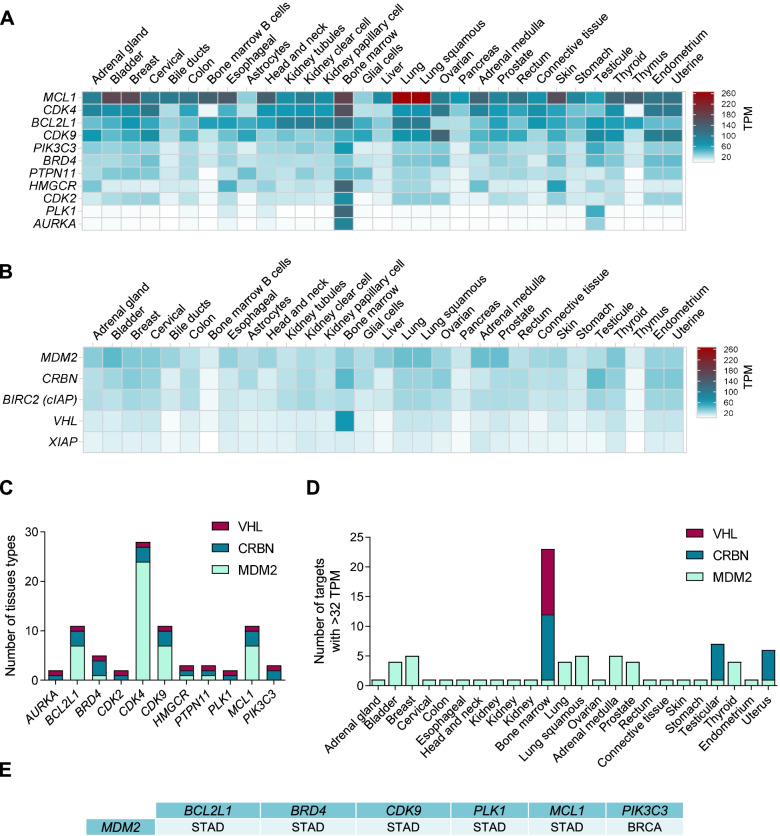
RNA expression in TPM of targets and ligases in several tissues. RNA expression in TPM of targets (**A**) and ligases (**B**) according to GEPIA2 data in several normal tissues. **C** Number of normal tissues in which ligases and targets are highly increased. **D** Number of targets, and ligases, with an expression greater than 32 TPM in the different tissues. **E** Ligases and targets expressed more than 32 TPM in tumor tissue, but less than 32 TPM in normal tissue. STAD: Stomach adenocarcinoma. BRCA: Breast invasive carcinoma

We observed that *MCL1* was one of the most highly expressed targets in normal tissues in different organs, especially in lung (Fig. 4A). Other targets highly presented included *BCL2L1, CDK4* and *CDK9.* When evaluating the presence of ligases, MDM2 was the most abundant in several tissues, followed by cIAP and CRBN. Of note a remarkable expression of VHL was observed in the bone marrow (Fig. 4B and C). Ligases with low expression in non-transformed tissue included *XIAP* and *VHL,* as shown in Fig. 4B and C. When associating ligases and targets, we observed that among all tissues evaluated *CDK4, CDK9, BCL2* and *MCL1* have a high expression in addition to *MDM2* (Fig. 4C). The tumor type that included more targets was Acute Myeloid Leukemia (AML) where CRBN and VHL were the most frequently expressed ligases (Fig. 4D).

Finally, we correlated the presence of ligases and targets within a tumor with the expression of that ligase in normal tissue. In this context the best-case scenario would be to identify a target and a ligase highly present in tumoral tissue, and the same ligase expressed in a low proportion in non-transformed tissue. Bearing this in mind we compared those that were higher expressed (more than 32 TPM) in tumor tissue but in a low proportion (less than 32 TPM) in normal tissue. With this approach we identified MDM2 ligase, coupled with inhibitors of the targets BCL2L1, BRD4, CDK9, PLK1 and MCL1 in stomach tumor tissue, as a therapeutic strategy. In a similar manner, the combination of MDM2 together with some PIK3C3 inhibitors in breast cancer was an attractive option.

## Discussion

In the present article we provide a snapshot of the current preclinical and clinical development state of PROTACs for the treatment of cancer. PROTACs are a novel family of agents that by binding to a protein of interest, are able to ubiquitin that target protein through the use of a ligand that binds to a E3 ubiquitin ligase [10, 11]. This process finally induces the degradation of the target by the proteasome [10, 11]. This strategy, that permits for the first time to degrade intracellular proteins, can act on classical oncogenic mediators like transcription factors (TFs) [11]. This approach has allowed the targeting of bromo and extra terminal domains (BETs) proteins like BRD2 or BRD4 or transcriptomic CDKs like CDK9, among others [10]. Selection of the right warhead and ubiquitin ligase ligand is key, considering that degradation of some of these target proteins in non-transformed tissue can induce severe toxicity given the fact that they are considered as pan-essential proteins or genes. Pan essential genes are those involved in fundamental pathways that are relevant for cell survival [15]. In this context, degradation of proteins that are coded by these genes can lead to severe side effects, if that degradation is not limited to the target tissue, and this can be a limitation for their clinical development.

In our work we analyzed the current landscape, observing that most compounds in development are using warhead ligands based on transcriptional inhibitors, kinase inhibitors or antiapoptotic agents. An interesting finding is the fact that agents in clinical development are those targeting the estrogen and androgen receptor in breast and prostate cancer, respectively [12]. Of note, targeting these two receptors enables to act exclusively on the tumor avoiding undesired toxicities, as these proteins are no expressed in non-transformed tissue.

When evaluating the effect that elimination of the identified genes would have on survival, we realized that some of these genes including *CDK9, AURKA* or *PLK1*, but also, although to a less extend, *BCL2, MCL1, PTPN11, BRD4, PTK2, MHGCR* or *PI3KC3*, could be considered as pan-essential genes, so inhibition of their presence in normal tissue would induce relevant side effects. In this context, degradation of these proteins could not be translated to the clinic due to the low therapeutic index. This limitation is not new in clinical development and has happened with chemical entities targeting CDK9 or AURKA. For these compounds the inadequate toxicity profile and low therapeutic index impaired the demonstration of clinical benefit [9]. As a proof of concept of this problem, some companies are developing AURKA inhibitors in nano-formulations with the aim to augment the therapeutic index by a selective delivery of the compound in the tumor [16].

When studding the presence of these targets we observed that BCL2 was highly expressed in lung and breast cancer and CDK4 in breast and prostate. Of note, MCL1 was extraordinary upregulated in lung followed by breast and gastric. These data suggest that the mentioned genes are potentially excellent targets in those indications. In parallel we explored the presence of ligases in the five more frequent tumors. Our idea was to evaluate in which tumors the specific use of a ligase ligand would produce a therapeutic advantage. The presence of ligases in specific tissues can increase the activity of PROTACs reducing the toxicities in other cells [17]. Indeed, this can be used to augment the anti-tumor activity in certain tumor types. We observed that MDM2 ligase could be used to create warheads ligand against BCL2L1, BRD4, CDK9, CDK4, PLK1, MCL1 and PIK3C3. When focused on specific indications, MDM2 could be used with MCL1 inhibitors in breast cancer and lung adenocarcinoma. This approach would enhance the therapeutic index in these two indications.

In this context, E3 tissue specificity can be incorporated to reduce on target dose-limiting toxicities, specifically if there is a lack of presence of that protein in a non-transformed tissue. An example was the development of BCL-XL PROTACs. BCL-XL inhibitors were not approved for the treatment of B cell lymphoma due to its on-target and dose limiting toxicity, mainly thrombocytopenia [18]. Since the VHL E3 ligase is poorly expressed in platelets, BCL-XL PROTACs targeted for degradation by that ligase did not induce thrombocytopenia, maintaining the same therapeutic efficacy as VHL that is expressed in the lymphomatous cells [18].

Within this framework, we analyzed the expression of ligases in non-transformed tissue. MDM2 was the most abundant in several tissues, followed by CRBN and VHL, which was especially highly in the bone marrow. Of note, ligases with low expression in non-transformed tissue included XIAP and VHL. Finally, we matched the presence of a target with the ligase. MDM2 ligase, coupled with inhibitors of the targets BCL2L1, BRD4, CDK9, PLK1 and MCL1 in stomach cancer, and MDM2 with PIK3C3 inhibitors in breast cancer seemed to be the best therapeutic construction.

Given the fact that this is an in silico approach our results are just estimations of what could potentially be used in the clinic. However, although with this limitation, optimization of clinical development using a bioinformatic study should be pursuit, to avoid the high attrition rate in cancer drug development.

The importance of this family of agents is demonstrated by the fact that some of these agents have entered clinical development. Table 1 describes the full list of early clinical stage PROTACs in which we aim to highlight two compounds ARV-110 and ARV-471 that target the estrogen and androgen receptor, respectively, and that are currently in phase II (Table 1).

**Table 1:** Clinical trials of PROTACs compounds, disease or tumor for which they are being tested, and clinical phase

NCT	Compound (Target)	Conditions	Phase
NCT03888612	ARV-110 (AR)	Prostate cancer metastatic	I/II
NCT04072952	ARV-471 (ER)	Breast cancer	I/II
NCT05067140	ARV-766 (AR)	Prostate cancer metastatic	I
NCT04428788	CC-94676 (AR)	Prostatic neoplasms	I
NCT04886622	DT2216 (BCL-xL)	Solid tumor/Hematologic malignancy	I
NCT04772885	KT-474 (IRAK4)	Healthy volunteer/atopic dermatitis/hidradenitis suppurativa	I
NCT04830137	NX-2127 (BTK)	Hematologic malignancy	I
NCT03891953	DKY709 (IKZF2)	Non-small-cell lung carcinoma/Melanoma/Nasopharyngeal carcinoma/Colorectal cancer/TNBC	I

In recent years, new types of PROTACs have been synthesized with covalent ligands that improve the selectivity and potency of the compounds. Indeed, the covalent targeting of E3 ligases is leading to the discovery of new ligands for novel E3 ligases such as DCAF16, RNF114 and RNF4. In addition, the different types of bindings, both with the target protein and with the E3 ligase, have been studied. Among them, the best option is to use a reversible binding with the protein of interest, thus maintaining the catalytic activity of the PROTAC, and a reversible or irreversible covalent binding with the E3 ligases. These PROTACs would be the solution for proteins for which only weak reversible ligands exist. Furthermore, it is relevant to mention that these covalently bound PROTACs are currently in preclinical development [19–21].

Finally, PROTACs could be exploited to degrade oncogenic mutated proteins for which resistance to chemical inhibitor has been developed [22, 23] In this context, several preclinical data suggests that resistance to kinase inhibitors can be overcome if the mutated oncoprotein is degraded with the use of a specific PROTAC. As mentioned before, ARV-471 and ARV-110, both PROTACS targeting the estrogen and androgen receptor, respectively, have shown preclinical activity in mutated models of the mentioned proteins, and particularly ARV-471 have showed clinical efficacy in a patient with a mutation at the estrogen receptor [24].

## Conclusions

In conclusion, our article demonstrates that an in silico analyses can rationally support the preclinical and clinical development of these compounds helping drug developers to design smart drugs for specific indications.

## Supplementary Information

**Additional file 1.**

## Data Availability

All data generated or analyzed during this study are included in this published article (and its supplementary information files).

## Abbreviations

ALK: Anaplastic lymphoma kinase
AML: Acute myeloid leukemia
AR: Androgen receptor
AURKA: Aurora kinase A
BCL2: B-cell lymphoma 2
BETs: Bromo and extra terminal domains
BRCA: Breast cancer
BRD4: Bromodomain-containing protein 4
CDK: Cyclin dependent kinase
COAD: Colon adenocarcinoma
CRBN: Cereblon
CRISPR: Clustered Regularly Interspaced Short Palindromic Repeats
EGFR: Epidermal growth factor receptor
HMGCR: 3-Hydroxy-3-Methylglutaryl-CoA Reductase
IAP: Islet amyloid polypeptide
LUAD: Lung adenocarcinoma
MCL1: Myeloid cell leukemia 1
MDM2: Murine double minute 2
PIK3C3: Phosphatidylinositol 3-kinase catalytic subunit type 3
PLK1: Polo-like kinase 1
PRAD: Prostate adenocarcinoma
PROTAC: Proteolysis targeting chimeras
PTPN11: Protein Tyrosine Phosphatase Non-Receptor Type 11
siRNA: Small interfering RNA
STAD: Stomach adenocarcinoma
TFs: Transcription factors
TPM: Transcript per million
VHL: Von Hippel-Lindau tumor suppressor
