# Supplementary material for: Clinical considerations for the design of PROTACs in cancer
Source: Mol Cancer. 2022 Mar 7;21:67. doi: 10.1186/s12943-022-01535-7 (PMC8900451; doi:10.1186/s12943-022-01535-7)
Supplement: Supplementary file 1 — Additional file 1. [file 12943_2022_1535_MOESM1_ESM.docx]

| **FUNCTION PROTEIN OF INTEREST (POI)** | **POI** | **LIGAND FOR E3 LIGASE** | **WARHEAD** | **PRECLINICAL TUMOR MODEL** | **REFERENCE** |
| --- | --- | --- | --- | --- | --- |
| Apoptotic mediator | BCL-xL | CRBN E3 ubiquitin ligase and VHL E3 ubiquitin ligase | Navitoclax | T-cell acute lymphoblastic leukemia cell line (MOLT-4) | (25) |
|  | BCL-xL | CRBN E3 ubiquitin ligase and VHL E3 ubiquitin ligase | ABT-263 | Cutaneous T-cell lymphoma cell line (MyLa 1929) | (26) |
|  | BCL-xL | VHL E3 ubiquitin ligase | A-1155463 | Leukemia cell lines (MOLT-4, THP-1) | (27) |
|  | BCL-XL | Von Hippel-Lindau (VHL) E3 ligase | ABT263 | Leukemia and cancer cells Several xenograft tumors | (18) |
|  | MCL1 | E3 ligase CUL4A−DDB1 CRBN E3 ligase |  | Acute myeloid leukemia | (28) |
| Mitotic kinase/Transcriptional regulator | Dual PLK1/BRD4 | CRBN E3 ligase-based BET PROTAC | BI2536 | Human acute leukemia MOLM-13 and KG1 cells, MV4-11 tumor xenograft model | (29) |
| Cytokines | TGF-β1 | Thalidomide CRBN E3 ubiquitin ligase | P144 | Leukemia (THP-1), lung adenocarcinoma (A549), Breast cancer (MCF-7), glioblastoma (U87), liver cancer (HepG2) cell lines | (30) |
| DNA repair | PARP-1 | Lenalidomide CRBN E3 ubiquitin ligase | Olaparib | Colorectal cancer (SW620) | (31) |
|  | PARP-1 | Thalidomide/Lenalidomide CRBN E3 ubiquitin ligase | Olaparib | Breast cancer (MDA-MB-436), pancreatic cancer (Capan-1) cell lines | (32) |
| Immune checkpoint | PD-L1 | Pomalidomide CRBN E3 ubiquitin ligase | BMS-1198 | Lung cancer (A549, H1299), breast cancer (MDA-BM-231), mouse skin melanoma (B16F10) cell lines | (33) |
| Metabolism of various substrates | CYP1B1 | Thalidomide CRBN E3 ubiquitin ligase | Derivative of ANF | Prostate cancer cell line (DU145) | (34) |
| Microtubule stabilization | Tau | VHL E3 ubiquitin ligase | Tau binder | HEK293 and SH-SY5Y | (35) |
| Metabolism | HMGCR | CRBN E3 ubiquitin ligase | Atorvastatin | Chinese hamster ovary cells (SRD15) | (36) |
| Protein kinase | AURKA | Thalidomide CRBN E3 ubiquitin ligase and two with HIF1-derived peptidomimetics VHL E3 ubiquitin ligase | Alisertib | Leukemia cell line (MV4-11) | (37) |
|  | CDK2/9 | Pomalidomide CRBN E3 ubiquitin ligase | AT-7519 and FN-1501 | PC-3 (Prostate cancer) | (38) |
|  | CDK4/6 | VHL, CRBN and IAP binders E3 ligase | Palbociclib | Leukemic T-cell lymphoblast cell line (Jurkat) | (39) |
|  | CDK9 | CRBN E3 ubiquitin ligase | BAY-1143572 | Leukemia (MV4-11, MOLM13), colorectal cancer (HCT116), Mouse skin melanoma (B16), lung adenocarcinoma (A549), breast cancer (MCF7) and liver cancer (HepG2) cell lines | (40) |
|  | GSK-3β | CRBN E3 ubiquitin ligase | Pyridinethiazole-based inhibitor G1 | Pheochromocytoma cell line (PC12) | (41) |
|  | IGF-1R and Src | Pomalidomide CRBN E3 ubiquitin ligase | N2-phenyl-N4-(1H-pyrazol-3-yl)pyrimidine-2,4-diamine | Breast cancer (MCF-7), lung carcinoma (A549) | (42) |
|  | IRAK4 | Pomalidomide CRBN E3 ubiquitin ligase | IRAK4 inhibitor 1 | Non-Hodgkin lymphoma (OCI-LY10) and diffuse large B cell lymphoma (TMD8) | (43) |
|  | JAK | IAP E3 ligase | Pyrimidine, quinoxaline | Leukemia monocytic cell line (THP-1) | (44) |
|  | MEK | VHL E3 ubiquitin ligase | MEK1 inhibitor | A375 Malignant melanoma | (45) |
|  | p38 α and β | Thalidomide CRBN E3 ubiquitin ligase | PH-797804 | Breast cancer (T47D, MDA-MB-231) cell lines | (46) |
|  | RIPK2 | IAP E3 ubiquitin ligase | Aminobenzothiazole-quinoline based RIPK2 binder | Leukemia monocytic cell line (THP-1) | (47) |
|  | ALK | Von Hippel-Lindau (VHL) E3 ligase | Ceritinib | ALK-positive NSCLC ALK fusion positive cell lines, SU-DHL-1 and H3122 | (48)  (49) |
|  | CDK6 | E3 ubiquitin ligase CRBN | Palbociclib | Acute myeloid leukemia cells | (50) |
|  | CDK6 | E3 ubiquitin ligase CRBN | Palbociclib | Pancreatic cancer | (50) |
|  | CDK9 | E3 ubiquitin ligase CRBN | Wogonin (natural product isolated from the Scutellaria baicalensis) | Breast cancer | (51) |
|  | CDK9 | E3 ubiquitin ligase CRBN |  | HCT116 cells. No explored anti-proliferative activity | (52) |
|  | SGK3 | E3 ubiquitin ligase CRBN | SGK inhibitors 308-R or 290R | ZR-75-1 and CAMA-1 cancer cell lines | (53) |
|  | FLT-3 |  | Quizartinib | Acute myeloid leukemia | (54) |
|  | MET and VEGFR | Von Hippel-Lindau (VHL) E3 ligase | Foretinib | MDA-MB-231 cell lines Triple negative breast cancer | (55) |
|  | FAk |  | Defactinib | Breast cancer cell lines | (56) |
|  | EGFR/MET | Von Hippel-Lindau (VHL) E3 ligase | Lapatinib, gefitinib, afatinib, foretinib | Cell lines: OVACR8, HeLa, HCC827, H3255, H1975, MDA-MB-231 | (57) |
|  | SGK3 and PIK3C3 | Cul2 Von Hippel-Lindau (VHL) E3 ligase VHL and cIAP ligands | Chloroalkane | Kidney cancer | (58) |
|  | c-ABL and BCR-ABL | CRBN or VHL E3 ligases | Bosutinib and dasatinib | K562 human chronic myelogenous leukemia cells | (59) |
|  | B-Raf | CRBN E3 ligases |  | Breast cancer cell line: MCF7 | (60) |
|  | CDK6 | E3 ubiquitin ligase CRBN | Palbociclib | Hematopoietic cancer cells including multiple myeloma and copy-amplified/mutated forms of CDK6 | (61) |
|  | ALK | VHL-1 E3 ligase | Brigatinib | Lymphoma cell line (SR) and lung cancer (H69. H1688) | (62) |
|  | ALK | CRBN E3 ubiquitin ligase | LDK378 | Lung cancer (H3122, H2228, H1299, A549), cervical cancer (HeLa) cell lines | (63) |
|  | ALK | CRBN E3 ubiquitin. Lenalidomide/thalidomide-based linkers | Alectinib | Lymphoma cell line (SR) | (64) |
| Protein phosphatase | SHP2 | VHL-1 E3 ubiquitin ligase | SHP2 inhibitor (derivative of SHP099) | human esophageal squamous carcinoma (KYSE520), leukemia (MV4;11) | (65) |
|  | SHP2 | Pomalidomide CRBN E3 ubiquitin ligase | SHP099 | Cervical cancer cell line (HeLa) | (66) |
|  | SHP2 | Thalidomide CRBN E3 ubiquitin ligase | TNO155 | Leukemia cell line (MV4-11) | (67) |
| Signal transducer protein | KRAS^G12C^ | IMiDs (thalidomide analog) CRBN E3 ubiquitin ligase | G12C-directed covalent quinazoline-based switch II pocket ligand | Pancreatic cancer cell line (MiaPaCa2) | (68) |
| Transcriptional regulator | AR | Low affinity VHL ligand (shorter linker) | AR antagonist | Prostate cancer cell lines (LNCaP, VCaP, 22Rv1) | (69) |
|  | AR | Small-molecule ligand that would bind to an E3 ubiquitin ligase (VHL) complex | ARI-16 | Prostate cancer cell lines (CWR-R1, LNCaP, 22Rv1) | (70) |
|  | AR | SARD derivative. Triazole backbone, HyT ligand | AR ligand | Stomach cancer (SGC-7901), breast cancer (MCF-7), normal gastric (GES-1), prostatic cancer (LNCaP) cell lines | (71) |
|  | AR | TD-106. CRBN E3 ubiquitin ligase | Tetramethylcyclobutane-based AR antagonist | Prostate cancer (LNCaP, VCaP) cell lines | (72) |
|  | AR | VHL-1 E3 ligase ligand | AR antagonist | Prostate cancer cell line (VCaP) | (73) |
|  | AR | . Thalidomide. CRBN E3 ubiquitin ligase | Nonsteroidal AR antagonist | Prostate cancer cell line (LNCaP) | (74) |
|  | AR-V7 and AR | CRL2VHL E3 ubiquitin ligase | AR's DNA binding domain | Prostate cancer cell lines (LNCaP, 22Rv1, VCaP, PC3, and DU145) | (75) |
|  | BRAF | Pomalidomide, thalidomide (CRBN E3 ubiquitin ligase), VH032 (VHL E3 ubiquitin ligase) | BI-882370 | Melanoma (A375), colorectal cancer (HCT116), breast cancer (MDA-MB-231) cell lines | (76) |
|  | BRD4 | Lenalidomide/pomalidomide CRBN E3 ubiquitin ligase | BRD4 inhibitor | Leukemia (HL-60), Burkitt's lymphoma (Raji), acute monocytic leukemia (THP-1) cell lines | (77) |
|  | BRD4 | VHL E3 ubiquitin ligase | JQ1 | Breast cancer cell lines (MCF-7, MDA-MB-231, SKBR3, BT474) | (78) |
|  | BRD4 | Lenalidomide CRBN E3 ubiquitin ligase | ABBV-075 | Pancreatic cancer cell line (BxPC3) | (79) |
|  | BRD4 (BD1) | Thalidomide CRBN E3 ubiquitin ligase | Selective BD1-inhibitor | Leukemia, Melanoma, | (80) |
|  |  |  |  | Non-Small Cell Lung Cancer, |  |
|  |  |  |  | Colon Cancer, |  |
|  |  |  |  | CNS Cancer, |  |
|  |  |  |  | Prostate cancer, |  |
|  |  |  |  | Ovarian Cancer, |  |
|  |  |  |  | Breast cancer, |  |
|  |  |  |  | Renal cancer |  |
|  | CBP and p300 | Pomalidomide CRBN E3 ubiquitin ligase | GNE-781 | Leukemia (HAP-1), multiple myeloma (MM1S) | (81) |
|  | CREPT | VHL E3 ubiquitin ligase | CREPT binding | Pancreatic cancer cell line (Panc-1) | (82) |
|  | EED (subunit PRC2) | VHL E3 ubiquitin ligase | EED ligand | Karpas422 Human B-Cell Non-Hodgkin Lymphoma | (83) |
|  | eEF2K | Thalidomide CRBN E3 ubiquitin ligase | A484954 | Breast cancer cell line (MDA-MB-231) | (84) |
|  | EGFR | Lenalidomide (CRBN E3 ubiquitin ligase) and VHL-L E3 ubiquitin ligase | EGFR-TKI | Lung adenocarcinoma cell line (HCC827) | (85) |
|  | EGFR | Lenalidomide CRBN E3 ubiquitin ligase | Osimertinib | Non-small cell lung cancer (PC9, H1975) cell lines | (86) |
|  | EGFR | Lenalidomide (CRBN E3 ubiquitin ligase) and VHL-L E3 ubiquitin ligase | Fourth-generation EGFR-TKI | Lung adenocarcinoma (HCC827, H1975) | (87) |
|  | EGFR ^L858R+T790M^ | Pomalidomide CRBN E3 ubiquitin ligase | Canertinib | Lung cancer (H1975, PC9Brca1) | (88) |
|  | EGFR^L858R/T790M^ | MDM2 and VHL-1 E3 ubiquitin ligase | XTF-262 | Non-small cell lung cancer (H1975), squamous carcinoma (A431) cell lines | (89) |
|  | ERα | HIF sequence: Leu-Ala-Pro(OH)-Tyr-Ile) from HIF-1α VHL E3 ubiquitin ligase | Lactam cyclic peptide as ERα binding ligand | MCF7 (Breast cancer) | (90) |
|  | ERα | XIAP and VHL E3 ubiquitin ligase | Endoxifen | MCF7 (Breast cancer) | (91) |
|  | EZH2 | CRBN E3 ubiquitin ligase | GSK126 | B-cell lymphoma cell line (WSU-DLCL-2) | (92) |
|  | HDAC1 and HDAC6 | Thalidomide CRBN E3 ubiquitin ligase | Panobinostat | Leukemia (HL-60), oral adenosquamous carcinoma (Cal27), glioblastoma (U87, U251) | (93) |
|  | HDAC6 | VHL E3 ligase and lenalidomide CRBN E3 ligase | Nexturastat A | MM.1S cell line Multiple myeloma | (94) |
|  | STAT3 | CRBN E3 ubiquitin ligase | SI-109 | Molm-16 Megakaryoblastic leukemia, SU-DHL-1 Lymphoma | (95) |
|  | β-catenin | ALAPYIP VHL E3 ubiquitin ligase | xStAx | Colorectal cancer (HCT116, SW480, LoVo) cell lines | (96) |
|  | SMARCA2 SMARCA4 | Von Hippel-Lindau (VHL) E3 ligase |  | Myeloid leukemia (AML) Acute myeloid leukemia cells | (97) |
|  | BCL6 | E3 ubiquitin ligase CRBN |  | Diffuse large B-cell lymphoma (DLBCL) Weak antiproliferative response | (98) |
|  | BET | E3 ubiquitin ligase CRBN: ARV-825 E3 ligase VHL: ARV-771 | OTX015 | Post-myeloproliferative neoplasm secondary (s) AML cells, Patient-derived cells, in vitro generated ruxolitinib-persister or ruxolitinib-resistant sAML cells, In vivo sAML mouse model | (29) |
|  | ERα | Von Hippel-Lindau (VHL) E3 ligase |  | ERα-positive breast cancer cells, MCF-7 mouse xenograft model | (99) |
|  | STAT3 | E3 ubiquitin ligase CRBN, Von Hippel-Lindau (VHL) E3 ligase | SI-109 and SI-108 | Acute myeloid leukemia and anaplastic large-cell lymphoma cell lines, multiple xenograft mouse models | (100) |
|  | AR | Von Hippel-Lindau (VHL) E3 ligase | Enzalutamide | Prostate cancer cell lines: VCaP, LNCaP, PC3. | (101) |
|  | ER | CRBN ligand/cullin 4A system, VHL ligand/Cullin 2 | ERD-308 | MCF-7 and T47D ER+ breast cancer cell lines | (99) |
| Chaperone | PDEδ | Pomalidomide CRBN E3 ubiquitin ligase | Deltazinone | Colorectal cancer (SW480, HCT116), pancreatic cancer (MiaPaca-2, Panc-1) | (102) |
| Na+/H+ exchangers | SLC9 | CRL CRBN E3 ubiquitin ligase | w9A | Breast cancer, colorectal cancer, leukemia, liver cancer, lung cancer, prostate cancer and skin cancer cell lines | (103) |
| Tyrosine kinase | BTK | IAP or cereblon E3 ligases | Ibrutinib |  | (104) |
|  | BTK | Phthalimide (CRBN E3 ubiquitin ligase) and VHL E3 ubiquitin ligase | BTK ligand | NAMALWA (Burkitt lymphoma) | (105) |
|  | BTK | Amine-functionalized E3 binder | Ibrutinib | B cell non-Hodgkin's lymphoma (Mino), Burkitt's lymphoma (Ramos) | (106) |
|  | BCR-ABL | Azobencene linked to lenalidomide (CRBN E3 ligase) | Dasatinib | Lung adenocarcinoma (A549), colon cancer (HCT116), breast cancer (MCF-7), kidney (HEK293T), bone marrow (K562) cell lines | (107) |
|  | BCR-ABL | Pomalidomide derivatives (CRBN E3 ligase) | Ponatinib, Asciminib | Murine cells (BaF3), leukemia cell line (K562) | (108) |
| Ubiquitination | MDM2 | CRBN E3 ubiquitin ligase | Nutlin | Leukemia cell lines (RS4; 11, MOLT-4 and NOMO-1) | (109) |
|  | MDM2 | Thalidomide CRBN E3 ubiquitin ligase | Ursolic acid | Lung cancer (A549), liver cancer (Huh7, HepG2) | (110) |
|  | ZFP91 | Pomalidomide CRBN E3 ubiquitin ligase | Napabucasin | Pancreatic cancer cell lines (MIAPaCa-2, BxPC-3) | (111) |
|  | MDM2 | CRBN ligands thalidomide and lenalidomide /cullin 4 E3 ligase complex | MD-224 | Leukemia cell lines, RS4;11 xenograft tumor model | (112) |

References

25. Zhang X, Thummuri D, Liu X, Hu W, Zhang P, Khan S, et al. Discovery of PROTAC BCL-X(L) degraders as potent anticancer agents with low on-target platelet toxicity. European journal of medicinal chemistry. 2020 Apr;192:112186.

26. Zhang X, He Y, Zhang P, Budamagunta V, Lv D, Thummuri D, et al. Discovery of IAP-recruiting BCL-X(L) PROTACs as potent degraders across multiple cancer cell lines. European journal of medicinal chemistry. 2020 Aug;199:112397.

27. Chung C-W, Dai H, Fernandez E, Tinworth CP, Churcher I, Cryan J, et al. Structural Insights into PROTAC-Mediated Degradation of Bcl-xL. ACS chemical biology. 2020 Sep;15(9):2316–23.

28. Papatzimas JW, Gorobets E, Maity R, Muniyat MI, MacCallum JL, Neri P, et al. From Inhibition to Degradation: Targeting the Antiapoptotic Protein Myeloid Cell Leukemia 1 (MCL1). Journal of medicinal chemistry. 2019 Jun;62(11):5522–40.

29. Saenz DT, Fiskus W, Qian Y, Manshouri T, Rajapakshe K, Raina K, et al. Novel BET protein proteolysis-targeting chimera exerts superior lethal activity than bromodomain inhibitor (BETi) against post-myeloproliferative neoplasm secondary (s) AML cells. Leukemia. 2017 Sep;31(9):1951–61.

30. Feng Y, Su H, Li Y, Luo C, Xu H, Wang Y, et al. Degradation of intracellular TGF-β1 by PROTACs efficiently reverses M2 macrophage induced malignant pathological events. Chemical communications (Cambridge, England). 2020 Mar;56(19):2881–4.

31. Zhang Z, Chang X, Zhang C, Zeng S, Liang M, Ma Z, et al. Identification of probe-quality degraders for Poly(ADP-ribose) polymerase-1 (PARP-1). Journal of enzyme inhibition and medicinal chemistry. 2020 Dec;35(1):1606–15.

32. Cao C, Yang J, Chen Y, Zhou P, Wang Y, Du W, et al. Discovery of SK-575 as a Highly Potent and Efficacious Proteolysis-Targeting Chimera Degrader of PARP1 for Treating Cancers. Journal of medicinal chemistry. 2020 Oct;63(19):11012–33.

33. Cheng B, Ren Y, Cao H, Chen J. Discovery of novel resorcinol diphenyl ether-based PROTAC-like molecules as dual inhibitors and degraders of PD-L1. European journal of medicinal chemistry. 2020 Aug;199:112377.

34. Zhou L, Chen W, Cao C, Shi Y, Ye W, Hu J, et al. Design and synthesis of α-naphthoflavone chimera derivatives able to eliminate cytochrome P450 (CYP)1B1-mediated drug resistance via targeted CYP1B1 degradation. European journal of medicinal chemistry. 2020 Mar;189:112028.

35. Wang W, Zhou Q, Jiang T, Li S, Ye J, Zheng J, et al. A novel small-molecule PROTAC selectively promotes tau clearance to improve cognitive functions in Alzheimer-like models. Theranostics. 2021;11(11):5279–95.

36. Li M-X, Yang Y, Zhao Q, Wu Y, Song L, Yang H, et al. Degradation versus Inhibition: Development of Proteolysis-Targeting Chimeras for Overcoming Statin-Induced Compensatory Upregulation of 3-Hydroxy-3-methylglutaryl Coenzyme A Reductase. Journal of medicinal chemistry. 2020 May;63(9):4908–28.

37. Adhikari B, Bozilovic J, Diebold M, Schwarz JD, Hofstetter J, Schröder M, et al. PROTAC-mediated degradation reveals a non-catalytic function of AURORA-A kinase. Nature chemical biology. 2020 Nov;16(11):1179–88.

38. Zhou F, Chen L, Cao C, Yu J, Luo X, Zhou P, et al. Development of selective mono or dual PROTAC degrader probe of CDK isoforms. European journal of medicinal chemistry. 2020 Feb;187:111952.

39. Anderson NA, Cryan J, Ahmed A, Dai H, McGonagle GA, Rozier C, et al. Selective CDK6 degradation mediated by cereblon, VHL, and novel IAP-recruiting PROTACs. Bioorganic & medicinal chemistry letters. 2020 May;30(9):127106.

40. Qiu X, Li Y, Yu B, Ren J, Huang H, Wang M, et al. Discovery of selective CDK9 degraders with enhancing antiproliferative activity through PROTAC conversion. European journal of medicinal chemistry. 2021 Feb;211:113091.

41. Jiang X, Zhou J, Wang Y, Liu X, Xu K, Xu J, et al. PROTACs suppression of GSK-3β, a crucial kinase in neurodegenerative diseases. European journal of medicinal chemistry. 2021 Jan;210:112949.

42. Manda S, Lee NK, Oh D-C, Lee J. Design, Synthesis, and Biological Evaluation of Proteolysis Targeting Chimeras (PROTACs) for the Dual Degradation of IGF-1R and Src. Molecules (Basel, Switzerland). 2020 Apr;25(8).

43. Chen Y, Ning Y, Bai G, Tong L, Zhang T, Zhou J, et al. Design, Synthesis, and Biological Evaluation of IRAK4-Targeting PROTACs. ACS medicinal chemistry letters. 2021 Jan;12(1):82–7.

44. Shah RR, Redmond JM, Mihut A, Menon M, Evans JP, Murphy JA, et al. Hi-JAK-ing the ubiquitin system: The design and physicochemical optimisation of JAK PROTACs. Bioorganic & medicinal chemistry. 2020 Mar;28(5):115326.

45. Vollmer S, Cunoosamy D, Lv H, Feng H, Li X, Nan Z, et al. Design, Synthesis, and Biological Evaluation of MEK PROTACs. Journal of medicinal chemistry. 2020 Jan;63(1):157–62.

46. Donoghue C, Cubillos-Rojas M, Gutierrez-Prat N, Sanchez-Zarzalejo C, Verdaguer X, Riera A, et al. Optimal linker length for small molecule PROTACs that selectively target p38α and p38β for degradation. European journal of medicinal chemistry. 2020 Sep;201:112451.

47. Mares A, Miah AH, Smith IED, Rackham M, Thawani AR, Cryan J, et al. Extended pharmacodynamic responses observed upon PROTAC-mediated degradation of RIPK2. Communications biology. 2020 Mar;3(1):140.

48. Kong X, Pan P, Sun H, Xia H, Wang X, Li Y, et al. Drug Discovery Targeting Anaplastic Lymphoma Kinase (ALK). Journal of medicinal chemistry. 2019 Dec;62(24):10927–54.

49. Kang CH, Lee DH, Lee CO, Du Ha J, Park CH, Hwang JY. Induced protein degradation of anaplastic lymphoma kinase (ALK) by proteolysis targeting chimera (PROTAC). Biochemical and biophysical research communications. 2018 Oct;505(2):542–7.

50. Brand M, Jiang B, Bauer S, Donovan KA, Liang Y, Wang ES, et al. Homolog-Selective Degradation as a Strategy to Probe the Function of CDK6 in AML. Cell chemical biology. 2019 Feb;26(2):300-306.e9.

51. Bian J, Ren J, Li Y, Wang J, Xu X, Feng Y, et al. Discovery of Wogonin-based PROTACs against CDK9 and capable of achieving antitumor activity. Bioorganic chemistry. 2018 Dec;81:373–81.

52. Robb CM, Contreras JI, Kour S, Taylor MA, Abid M, Sonawane YA, et al. Chemically induced degradation of CDK9 by a proteolysis targeting chimera (PROTAC). Chemical communications (Cambridge, England). 2017 Jul;53(54):7577–80.

53. Tovell H, Testa A, Zhou H, Shpiro N, Crafter C, Ciulli A, et al. Design and Characterization of SGK3-PROTAC1, an Isoform Specific SGK3 Kinase PROTAC Degrader. ACS chemical biology. 2019 Sep;14(9):2024–34.

54. Burslem GM, Song J, Chen X, Hines J, Crews CM. Enhancing Antiproliferative Activity and Selectivity of a FLT-3 Inhibitor by Proteolysis Targeting Chimera Conversion. Journal of the American Chemical Society. 2018 Dec;140(48):16428–32.

55. Bondeson DP, Smith BE, Burslem GM, Buhimschi AD, Hines J, Jaime-Figueroa S, et al. Lessons in PROTAC Design from Selective Degradation with a Promiscuous Warhead. Cell chemical biology. 2018 Jan;25(1):78-87.e5.

56. Cromm PM, Samarasinghe KTG, Hines J, Crews CM. Addressing Kinase-Independent Functions of Fak via PROTAC-Mediated Degradation. Journal of the American Chemical Society. 2018 Dec;140(49):17019–26.

57. Burslem GM, Smith BE, Lai AC, Jaime-Figueroa S, McQuaid DC, Bondeson DP, et al. The Advantages of Targeted Protein Degradation Over Inhibition: An RTK Case Study. Cell chemical biology. 2018 Jan;25(1):67-77.e3.

58. Tovell H, Testa A, Maniaci C, Zhou H, Prescott AR, Macartney T, et al. Rapid and Reversible Knockdown of Endogenously Tagged Endosomal Proteins via an Optimized HaloPROTAC Degrader. ACS chemical biology. 2019 May;14(5):882–92.

59. Lai AC, Toure M, Hellerschmied D, Salami J, Jaime-Figueroa S, Ko E, et al. Modular PROTAC Design for the Degradation of Oncogenic BCR-ABL. Angewandte Chemie (International ed in English). 2016 Jan;55(2):807–10.

60. Chen H, Chen F, Pei S, Gou S. Pomalidomide hybrids act as proteolysis targeting chimeras: Synthesis, anticancer activity and B-Raf degradation. Bioorganic chemistry. 2019 Jun;87:191–9.

61. Su S, Yang Z, Gao H, Yang H, Zhu S, An Z, et al. Potent and Preferential Degradation of CDK6 via Proteolysis Targeting Chimera Degraders. Journal of medicinal chemistry. 2019 Aug;62(16):7575–82.

62. Sun N, Ren C, Kong Y, Zhong H, Chen J, Li Y, et al. Development of a Brigatinib degrader (SIAIS117) as a potential treatment for ALK positive cancer resistance. European journal of medicinal chemistry. 2020 May;193:112190.

63. Yan G, Zhong X, Yue L, Pu C, Shan H, Lan S, et al. Discovery of a PROTAC targeting ALK with in vivo activity. European journal of medicinal chemistry. 2021 Feb;212:113150.

64. Ren C, Sun N, Kong Y, Qu X, Liu H, Zhong H, et al. Structure-based discovery of SIAIS001 as an oral bioavailability ALK degrader constructed from Alectinib. European journal of medicinal chemistry. 2021 May;217:113335.

65. Wang M, Lu J, Wang M, Yang C-Y, Wang S. Discovery of SHP2-D26 as a First, Potent, and Effective PROTAC Degrader of SHP2 Protein. Journal of medicinal chemistry. 2020 Jul;63(14):7510–28.

66. Zheng M, Liu Y, Wu C, Yang K, Wang Q, Zhou Y, et al. Novel PROTACs for degradation of SHP2 protein. Bioorganic chemistry. 2021 May;110:104788.

67. Yang X, Wang Z, Pei Y, Song N, Xu L, Feng B, et al. Discovery of thalidomide-based PROTAC small molecules as the highly efficient SHP2 degraders. European journal of medicinal chemistry. 2021 Jun;218:113341.

68. Zeng M, Xiong Y, Safaee N, Nowak RP, Donovan KA, Yuan CJ, et al. Exploring Targeted Degradation Strategy for Oncogenic KRAS(G12C). Cell chemical biology. 2020 Jan;27(1):19-31.e6.

69. Han X, Zhao L, Xiang W, Qin C, Miao B, Xu T, et al. Discovery of Highly Potent and Efficient PROTAC Degraders of Androgen Receptor (AR) by Employing Weak Binding Affinity VHL E3 Ligase Ligands. Journal of medicinal chemistry. 2019 Dec;62(24):11218–31.

70. Kregel S, Wang C, Han X, Xiao L, Fernandez-Salas E, Bawa P, et al. Androgen receptor degraders overcome common resistance mechanisms developed during prostate cancer treatment. Neoplasia (New York, NY). 2020 Feb;22(2):111–9.

71. Xie H, Liang J-J, Wang Y-L, Hu T-X, Wang J-Y, Yang R-H, et al. The design, synthesis and anti-tumor mechanism study of new androgen receptor degrader. European journal of medicinal chemistry. 2020 Oct;204:112512.

72. Takwale AD, Jo S-H, Jeon YU, Kim HS, Shin CH, Lee HK, et al. Design and characterization of cereblon-mediated androgen receptor proteolysis-targeting chimeras. European journal of medicinal chemistry. 2020 Dec;208:112769.

73. Chen L, Han L, Mao S, Xu P, Xu X, Zhao R, et al. Discovery of A031 as effective proteolysis targeting chimera (PROTAC) androgen receptor (AR) degrader for the treatment of prostate cancer. European journal of medicinal chemistry. 2021 Apr;216:113307.

74. Kim G-Y, Song CW, Yang Y-S, Lee N-R, Yoo H-S, Son SH, et al. Chemical Degradation of Androgen Receptor (AR) Using Bicalutamide Analog-Thalidomide PROTACs. Molecules (Basel, Switzerland). 2021 Apr;26(9).

75. Lee GT, Nagaya N, Desantis J, Madura K, Sabaawy HE, Kim W-J, et al. Effects of MTX-23, a Novel PROTAC of Androgen Receptor Splice Variant-7 and Androgen Receptor, on CRPC Resistant to Second-Line Antiandrogen Therapy. Molecular cancer therapeutics. 2021 Mar;20(3):490–9.

76. Posternak G, Tang X, Maisonneuve P, Jin T, Lavoie H, Daou S, et al. Functional characterization of a PROTAC directed against BRAF mutant V600E. Nature chemical biology. 2020 Nov;16(11):1170–8.

77. Zhang F, Wu Z, Chen P, Zhang J, Wang T, Zhou J, et al. Discovery of a new class of PROTAC BRD4 degraders based on a dihydroquinazolinone derivative and lenalidomide/pomalidomide. Bioorganic & medicinal chemistry. 2020 Jan;28(1):115228.

78. Maneiro MA, Forte N, Shchepinova MM, Kounde CS, Chudasama V, Baker JR, et al. Antibody-PROTAC Conjugates Enable HER2-Dependent Targeted Protein Degradation of BRD4. ACS chemical biology. 2020 Jun;15(6):1306–12.

79. Zhang J, Chen P, Zhu P, Zheng P, Wang T, Wang L, et al. Development of small-molecule BRD4 degraders based on pyrrolopyridone derivative. Bioorganic chemistry. 2020 Jun;99:103817.

80. Jiang F, Wei Q, Li H, Li H, Cui Y, Ma Y, et al. Discovery of novel small molecule induced selective degradation of the bromodomain and extra-terminal (BET) bromodomain protein BRD4 and BRD2 with cellular potencies. Bioorganic & medicinal chemistry. 2020 Jan;28(1):115181.

81. Vannam R, Sayilgan J, Ojeda S, Karakyriakou B, Hu E, Kreuzer J, et al. Targeted degradation of the enhancer lysine acetyltransferases CBP and p300. Cell chemical biology. 2021 Apr;28(4):503-514.e12.

82. Ma D, Zou Y, Chu Y, Liu Z, Liu G, Chu J, et al. A cell-permeable peptide-based PROTAC against the oncoprotein CREPT proficiently inhibits pancreatic cancer. Theranostics. 2020;10(8):3708–21.

83. Hsu JH-R, Rasmusson T, Robinson J, Pachl F, Read J, Kawatkar S, et al. EED-Targeted PROTACs Degrade EED, EZH2, and SUZ12 in the PRC2 Complex. Cell chemical biology. 2020 Jan;27(1):41-46.e17.

84. Liu Y, Zhen Y, Wang G, Yang G, Fu L, Liu B, et al. Designing an eEF2K-Targeting PROTAC small molecule that induces apoptosis in MDA-MB-231 cells. European journal of medicinal chemistry. 2020 Oct;204:112505.

85. Zhang H, Zhao H-Y, Xi X-X, Liu Y-J, Xin M, Mao S, et al. Discovery of potent epidermal growth factor receptor (EGFR) degraders by proteolysis targeting chimera (PROTAC). European journal of medicinal chemistry. 2020 Mar;189:112061.

86. He K, Zhang Z, Wang W, Zheng X, Wang X, Zhang X. Discovery and biological evaluation of proteolysis targeting chimeras (PROTACs) as an EGFR degraders based on osimertinib and lenalidomide. Bioorganic & medicinal chemistry letters. 2020 Jun;30(12):127167.

87. Zhao H-Y, Yang X-Y, Lei H, Xi X-X, Lu S-M, Zhang J-J, et al. Discovery of potent small molecule PROTACs targeting mutant EGFR. European journal of medicinal chemistry. 2020 Dec;208:112781.

88. Qu X, Liu H, Song X, Sun N, Zhong H, Qiu X, et al. Effective degradation of EGFR(L858R+T790M) mutant proteins by CRBN-based PROTACs through both proteosome and autophagy/lysosome degradation systems. European journal of medicinal chemistry. 2021 Jun;218:113328.

89. Zhang X, Xu F, Tong L, Zhang T, Xie H, Lu X, et al. Design and synthesis of selective degraders of EGFR(L858R/T790M) mutant. European journal of medicinal chemistry. 2020 Apr;192:112199.

90. Dai Y, Yue N, Gong J, Liu C, Li Q, Zhou J, et al. Development of cell-permeable peptide-based PROTACs targeting estrogen receptor α. European journal of medicinal chemistry. 2020 Feb;187:111967.

91. Dragovich PS, Adhikari P, Blake RA, Blaquiere N, Chen J, Cheng Y-X, et al. Antibody-mediated delivery of chimeric protein degraders which target estrogen receptor alpha (ERα). Bioorganic & medicinal chemistry letters. 2020 Feb;30(4):126907.

92. Liu Z, Hu X, Wang Q, Wu X, Zhang Q, Wei W, et al. Design and Synthesis of EZH2-Based PROTACs to Degrade the PRC2 Complex for Targeting the Noncatalytic Activity of EZH2. Journal of medicinal chemistry. 2021 Mar;64(5):2829–48.

93. Sinatra L, Bandolik JJ, Roatsch M, Sönnichsen M, Schoeder CT, Hamacher A, et al. Hydroxamic Acids Immobilized on Resins (HAIRs): Synthesis of Dual-Targeting HDAC Inhibitors and HDAC Degraders (PROTACs). Angewandte Chemie (International ed in English). 2020 Dec;59(50):22494–9.

94. Yang H, Lv W, He M, Deng H, Li H, Wu W, et al. Plasticity in designing PROTACs for selective and potent degradation of HDAC6. Chemical communications (Cambridge, England). 2019 Dec;55(98):14848–51.

95. Zhou H, Bai L, Xu R, Zhao Y, Chen J, McEachern D, et al. Structure-Based Discovery of SD-36 as a Potent, Selective, and Efficacious PROTAC Degrader of STAT3 Protein. Journal of medicinal chemistry. 2019 Dec;62(24):11280–300.

96. Liao H, Li X, Zhao L, Wang Y, Wang X, Wu Y, et al. A PROTAC peptide induces durable β-catenin degradation and suppresses Wnt-dependent intestinal cancer. Cell discovery. 2020;6:35.

97. Farnaby W, Koegl M, Roy MJ, Whitworth C, Diers E, Trainor N, et al. BAF complex vulnerabilities in cancer demonstrated via structure-based PROTAC design. Nature chemical biology. 2019 Jul;15(7):672–80.

98. McCoull W, Cheung T, Anderson E, Barton P, Burgess J, Byth K, et al. Development of a Novel B-Cell Lymphoma 6 (BCL6) PROTAC To Provide Insight into Small Molecule Targeting of BCL6. ACS chemical biology. 2018 Nov;13(11):3131–41.

99. Jiang Y, Deng Q, Zhao H, Xie M, Chen L, Yin F, et al. Development of Stabilized Peptide-Based PROTACs against Estrogen Receptor α. ACS chemical biology. 2018 Mar;13(3):628–35.

100. Bai L, Zhou H, Xu R, Zhao Y, Chinnaswamy K, McEachern D, et al. A Potent and Selective Small-Molecule Degrader of STAT3 Achieves Complete Tumor Regression In Vivo. Cancer cell. 2019 Nov;36(5):498-511.e17.

101. Salami J, Alabi S, Willard RR, Vitale NJ, Wang J, Dong H, et al. Androgen receptor degradation by the proteolysis-targeting chimera ARCC-4 outperforms enzalutamide in cellular models of prostate cancer drug resistance. Communications Biology [Internet]. 2018;1(1):100. Available from: https://doi.org/10.1038/s42003-018-0105-8

102. Cheng J, Li Y, Wang X, Dong G, Sheng C. Discovery of Novel PDEδ Degraders for the Treatment of KRAS Mutant Colorectal Cancer. Journal of medicinal chemistry. 2020 Jul;63(14):7892–905.

103. Bensimon A, Pizzagalli MD, Kartnig F, Dvorak V, Essletzbichler P, Winter GE, et al. Targeted Degradation of SLC Transporters Reveals Amenability of Multi-Pass Transmembrane Proteins to Ligand-Induced Proteolysis. Cell chemical biology. 2020 Jun;27(6):728-739.e9.

104. Tinworth CP, Lithgow H, Dittus L, Bassi ZI, Hughes SE, Muelbaier M, et al. PROTAC-Mediated Degradation of Bruton’s Tyrosine Kinase Is Inhibited by Covalent Binding. ACS chemical biology. 2019 Mar;14(3):342–7.

105 Jaime-Figueroa S, Buhimschi AD, Toure M, Hines J, Crews CM. Design, synthesis and biological evaluation of Proteolysis Targeting Chimeras (PROTACs) as a BTK degraders with improved pharmacokinetic properties. Bioorganic & medicinal chemistry letters. 2020 Feb;30(3):126877.

106. Gabizon R, Shraga A, Gehrtz P, Livnah E, Shorer Y, Gurwicz N, et al. Efficient Targeted Degradation via Reversible and Irreversible Covalent PROTACs. Journal of the American Chemical Society. 2020 Jul;142(27):11734–42.

107. Jin Y-H, Lu M-C, Wang Y, Shan W-X, Wang X-Y, You Q-D, et al. Azo-PROTAC: Novel Light-Controlled Small-Molecule Tool for Protein Knockdown. Journal of medicinal chemistry. 2020 May;63(9):4644–54.

108. Yang Y, Gao H, Sun X, Sun Y, Qiu Y, Weng Q, et al. Global PROTAC Toolbox for Degrading BCR-ABL Overcomes Drug-Resistant Mutants and Adverse Effects. Journal of medicinal chemistry. 2020 Aug;63(15):8567–83.

109. Wang B, Liu J, Tandon I, Wu S, Teng P, Liao J, et al. Development of MDM2 degraders based on ligands derived from Ugi reactions: Lessons and discoveries. European journal of medicinal chemistry. 2021 Jul;219:113425.

110. Qi Z, Yang G, Deng T, Wang J, Zhou H, Popov SA, et al. Design and linkage optimization of ursane-thalidomide-based PROTACs and identification of their targeted-degradation properties to MDM2 protein. Bioorganic chemistry. 2021 Jun;111:104901.

111. Hanafi M, Chen X, Neamati N. Discovery of a Napabucasin PROTAC as an Effective Degrader of the E3 Ligase ZFP91. Journal of medicinal chemistry. 2021 Feb;64(3):1626–48.

112 Li Y, Yang J, Aguilar A, McEachern D, Przybranowski S, Liu L, et al. Discovery of MD-224 as a First-in-Class, Highly Potent, and Efficacious Proteolysis Targeting Chimera Murine Double Minute 2 Degrader Capable of Achieving Complete and Durable Tumor Regression. Journal of medicinal chemistry. 2019 Jan;62(2):448–66.
